# Registry-based cohort study comparing percentages of patients reaching PASS for knee function outcomes after revision ACLR compared to primary ACLR

**DOI:** 10.1136/bmjopen-2023-081688

**Published:** 2024-08-09

**Authors:** Zachary J Herman, Janina Kaarre, Alberto Grassi, Eric Hamrin Senorski, Volker Musahl, Kristian Samuelsson

**Affiliations:** 1 Orthopaedic Surgery, University of Pittsburgh, Pittsburgh, Pennsylvania, USA; 2 Orthopaedics, Göteborgs Universitet Institutionen för Kliniska Vetenskaper, Goteborg, Sweden; 3 IRCCS Istituto Ortopedico Rizzoli, Bologna, Italy; 4 Health and Rehabilitation, Goteborgs Universitet, Goteborg, Sweden

**Keywords:** Knee, Musculoskeletal disorders, Orthopaedic sports trauma, Adult orthopaedics, SPORTS MEDICINE

## Abstract

**ABSTRACT:**

**Objectives:**

Reaching the Patient-Acceptable Symptom State (PASS) threshold for the Knee injury and Osteoarthritis Outcome Score (KOOS) has previously been reported to successfully identify individuals experiencing clinical success after anterior cruciate ligament reconstruction (ACLR). Thus, the objectives of this study were to examine and compare the percentages of patients meeting PASS thresholds for the different KOOS subscales 1 year postoperatively after primary ACLR compared with revision ACLR (rACLR) and multiply revised ACLR (mrACLR), and second, to examine the predictors for reaching PASS for KOOS Quality of Life (QoL) and Function in Sport and Recreation (Sport/Rec) after mrACLR.

**Design:**

Prospective observational registry study.

**Setting:**

The data used in this study was obtained from the Swedish National Ligament Registry and collected between 2005 and 2020.

**Participants:**

The study sample was divided into three different groups: (1) primary ACLR, (2) rACLR and (3) mrACLR. Data on patient demographic, injury and surgical characteristics were obtained as well as mean 1-year postoperative scores for KOOS subscales and the per cent of patients meeting PASS for each subscale. Additionally, the predictors of reaching PASS for KOOS Sport/Rec, and QoL subscales were evaluated in patients undergoing mrACLR.

**Results:**

Of the 22 928 patients included in the study, 1144 underwent rACLR and 36 underwent mrACLR. Across all KOOS subscales, the percentage of patients meeting PASS thresholds was statistically lower for rACLR compared with primary ACLR (KOOS Symptoms 22.5% vs 32.9%, KOOS Pain 84.9% vs 92.9%, KOOS Activities of Daily Living 23.5% vs 31.4%, KOOS Sport/Rec 26.3% vs 45.6%, KOOS QoL 26.9% vs 51.4%). Percentages of patients reaching PASS thresholds for all KOOS subscales were comparable between patients undergoing rACLR versus mrACLR. No predictive factors were found to be associated with reaching PASS for KOOS QoL and KOOS Sport/Rec 1 year postoperatively after mrACLR.

**Conclusion:**

Patients undergoing ACLR in the revision setting had lower rates of reaching acceptable symptom states for functional knee outcomes than those undergoing primary ACLR.

**Level of evidence:**

Prospective observational registry study, level of evidence II.

**Strengths and limitations of this study**

- The large sample size allows assessment of outcomes following anterior cruciate ligament (ACL) reconstruction among different ages, sexes and ethnicities.
- Considering the high coverage rate of ACL injuries in the registry, the study sample can be considered representative of the Swedish population.
- The results of the study may not be generalisable to other countries where different injury mechanisms and treatment approaches may be more common.

## Introduction

 As the number of anterior cruciate ligament reconstructions (ACLR) increases, the incidence of revision ACLR (rACLR) continues to rise, with rates as high as 15%–25% after primary ACLR.^1^,^3^ Patient-reported knee function and rate of return to sport after rACLR is widely accepted as inferior to that following the primary procedure,^4 5^ yet, less data exists on the multiply revised ACLR (mrACLR) and how its outcomes compare to rACLR.

The Patient-Acceptable Symptom State (PASS) threshold is a single-item treatment-response criteria which requires that a patient consider all aspects of life to determine whether the state of his or her joint is satisfactory after an intervention.^6^ It was developed through an anchor method where one, ‘simple’ question on acceptable knee function is linked to a patient-reported outcome to establish cut-off values that determine the clinical relevance of a treatment’s effect.^7^ These values have been developed for several patient-reported outcomes following various surgical procedures, including ACLR.^8^ In fact, reaching the PASS threshold for Knee injury and Osteoarthritis Outcome Score (KOOS) has successfully identified individuals who have experienced clinical success after ACLR with high sensitivity.^9^ Vega *et al* defined clinical success in their study as ‘changes in preoperative scores on the KOOS Pain subscale and the KOOS Knee Related Quality of Life (QoL) subscale in excess of minimally important difference or final KOOS Pain or QoL subscale scores in excess of previously defined PASS thresholds’*.*^9^ A 2020 ACLR consensus group concluded that measurement of PASS is valuable in the assessment of outcomes after ACLR and should be a priority, as the question of whether a patient perceives an acceptable symptom state is important.^10^ Furthermore, it is agreed that PASS values provide a tool to assist clinicians in the interpretation of patient-reported outcomes,^10^ thus PASS values can assist clinical decision-making. Numerous studies are present in the literature reporting predictive factors and percentages of patients meeting PASS thresholds after primary ACLR for the different KOOS subscales.^911^,^15^ However, less data exists exploring the predictors and percentages of patients meeting PASS for the different KOOS subscales in the rACLR setting. Thus, this information would be particularly important for a better understanding of different subjective patient outcomes and counselling patients before their respective surgery about their future subjective Quality of Life (QoL) and knee function.

As such, the primary purpose of the study was to examine and compare the percentages of patients meeting PASS thresholds for the different KOOS subscales 1 year postoperatively after primary ACLR compared with rACLR and mrACLR. Secondarily, it sought to examine the injury-, patient- and surgery-related predictors for reaching PASS for KOOS QoL and Function in Sport and Recreation (Sport/Rec) after mrACLR as these subscales have previously been recognised as viable subscales for assessing knee function and QoL in patient undergoing ACLR.^16 17^

## Methods

The Strengthening the Reporting of Observational Studies in Epidemiology guidelines were used to present this study.^18^

The data used in this study was obtained from the Swedish National Knee Ligament Registry (SNKLR), which covers information on patients who have undergone ACLR in Sweden. The registry boasts a coverage rate of over 90% and was created in January 2005. The data collected in the SNKLR includes information reported by both the patient and surgeon, such as demographical data, injury and surgical characteristics and patient-reported outcome measures. Surgeons report all demographical data, injury and surgery-related data, while patients complete questionnaires regarding their current knee function at 1-, 2-, 5- and 10-year follow-ups, including the KOOS. Information regarding ACL revision surgery is recorded separately and subsequently combined with data on primary ACLR using the patient’s social security numbers. The registry has been described in more detail in previous literature.^19 20^

### Data collection and study sample

This study included patients who underwent primary ACLR between 2005 and 2020 and were at least 15 years old at the time of surgery, with 1-year follow-up outcome data. Patients who had previously undergone knee surgery, had a concomitant fracture or had a concomitant posterior cruciate ligament or neurovascular injury were excluded. In addition, less commonly used femoral (staple, AO screw, retro screw, metal interference screw with Endopearl backup fixation, Graftmax, XO-button, ‘other’) and tibial fixation types (cobra, staple, Endobutton, Mitek anchor, ‘other’) were excluded. The study sample was divided into three different groups: (1) primary ACLR (patients undergoing only one ipsilateral ACLR surgery), (2) rACLR (patients undergoing only one ipsilateral rACLR surgery) and (3) mrACLR (patients undergoing at least two ipsilateral rACLR surgeries).

Data on patient demographical (age, body mass index (BMI), sex), injury (other concomitant injuries, activity at the time of injury) and surgical characteristics (time from injury to surgery, fixation device, ACL graft type) were additionally obtained from the registry. The activity at the time of injury was divided into six different groups: (1) alpine/skiing; (2) pivoting sport (American football/rugby, basketball, dancing, floorball, gymnastics, handball, ice hockey/bandy, martial arts, racket sports, soccer, volleyball, wrestling); (3) non-pivoting sport (cross-country skiing, cycling, horseback riding, motocross/endure, skateboarding, snowboarding and surfing/wakeboarding); (4) other physical activity (other recreational sport, exercise, trampoline); (5) traffic-related and (6) other (other outdoor activity and work). The tibial fixation devices were categorised into different groups, with six different subcategories for tibial fixation devices: (1) interference screw (metal screw, metal screw with backup staple fixation, resorbable screw, resorbable screw with backup post-fixation, metal screw with backup osteosuture, intrafix); (2) intratunnel fixation (rigidfix); (3) suture post (AO screw, suture washer); (4) retroscrew; (5) fixed suspensory fixation (retrobutton); (6) adjustable suspensory fixation (tightrope), and four different subcategories for femoral fixation devices: (1) fixed suspensory fixation (endobutton, retrobutton, ezloc); (2) intratunnel fixation (rigidfix, transfix); (3) interference screw (metal screw); (4) adjustable suspensory fixation (toggleloc, ultrabutton).

### Outcome measures

The primary outcome of interest was the 1-year postoperative PASS for KOOS subscales, thus, the 1- and 2-year KOOS outcomes were considered the same follow-up data.^21^ Each subscale is scored from 0 to 100, with a higher score indicating better outcomes.^22^ The responses are rated on a 5-point Likert Scale ranging from 0 to 4. While the KOOS was initially designed for knee osteoarthritis, it has also been used in other orthopaedic knee injuries/conditions, including ACLR, to assess outcomes following a surgical treatment.^10 22^ Additionally, the predictors for KOOS Sport/Rec and QoL subscales were evaluated in patients undergoing mrACLR. The KOOS questionnaire comprises five subscales, namely Pain, Symptoms, Sport/Rec, QoL and Activities of Daily Living (ADL). To exceed the PASS cut-offs was defined as achieving a PASS indicating that the patient is satisfied with their current symptom state. The PASS cut-offs (sensitivity; specificity) for patients undergoing ACLR have been previously reported to be: 88.9 (0.82; 0.81) for the KOOS Pain, 57.1 (0.78; 0.67) for the KOOS Symptoms, 100.0 (0.70; 0.89) for the KOOS ADL, 75.0 (0.87; 0.88) for the KOOS Sport/Rec and 62.5 (0.82; 0.85) for the KOOS QoL.^8^ The PASS cut-offs were determined by asking the study subjects to answer the question, ‘Taking into account all the activity you have during your daily life, your level of pain, and also your activity limitations and participation restrictions, do you consider the current state of your knee satisfactory?’.^8^

### Statistical analyses

All statistical analyses were performed by using SAS System for Windows software (V.9.4, SAS Institute, Cary, North Carolina, USA). Count (n) and proportion (%) were used to present categorical variables, while mean with SD and median with range were used for presenting continuous and ordinal data, respectively. For comparisons between groups χ^2^ test was used for non-ordered categorical variables and the Kruskal-Wallis test was used for continuous variables. For pairwise comparisons between groups, the Fisher’s exact test (two-sided) was used due to dichotomous variables, while χ^2^ test was used for non-ordered categorical variables. Fisher’s non-parametric permutation test was used for continuous variables. Finally, univariable logistic regression analyses were used for predictor analyses and presented as OR and area under the receiver operating characteristic. The level of significance was set to 0.05.

## Results

### Baseline characteristics

Out of the 22 928 patients who were included in the study, 21 748 underwent primary ACLR, 1144 underwent rACLR and 36 underwent mrACLR (table 1). The age at the time of the index ACLR was significantly different between the groups, with patients who underwent rACLR and mrACLR being significantly younger than patients who had only undergone primary ACLR (23.0 vs 22.3 vs 29.0; p=0.002). Additionally, patients who underwent mrACLR had significantly shorter mean time from primary ACLR to first revision than those patients undergoing rACLR (2.34 years vs 3.27 years; p=0.032). Finally, a greater proportion of patients undergoing rACLR had lateral meniscus injuries at the time of the index ACLR compared with those who underwent primary ACLR (27.4% vs 23.9%; p=0.0043). Baseline characteristics prior to the latest ACLR surgery can be found in online supplemental table S1.

**Table 1:** Baseline characteristics prior to the index ACLR

Variable	Total(n=22 928)	Only one ACLR (n=21 748)	One revision ACLR(n=1144)	Multiple revision ACLR(n=36)	P value	Test between groups p value		
						Only one ACLR vs one revision ACLR	Only one ACLR vs more than one revision ACLR	One revision ACLR vs more than one revision ACLR
Age at the time of surgery (years)	28.7±10.626 (16–74)	29.1±10.726 (16–74)	22.9±7.620 (16–56)	22.6±7.220.5 (16–44)	**<0.0001**	**0.0020**	**0.0020**	0.86
Sex (male)	12 055 (52.6)	11 429 (52.6)	604 (52.8)	22 (61.1)	0.58			
BMI (kg/m^2^)	24.7±3.324.2 (15.1–49.8)	24.7±3.324.2 (15.1–49.8)	24.5±3.124.2 (17.6; 37.6)	24.7±3.223.5 (19.6–34)	0.42			
Time from index ACLR to first revision (years)	3.25±2.492.39 (0.02–14.44)		3.27±2.512.44 (0.02–14.44)	2.34±1.631.75 (0.77–6.54)	**0.032**			
Time from index ACLR to last revision (years)	3.35±2.572.53 (0–14.44)		3.26±2.512.43 (0–14.44)	6.07±2.995.75 (0.97–13.33)	**<0.0001**			
Time from injury to index surgery (months)	20.4±36.68.3 (0–551)	20.9±37.38.5 (0–551)	11.3±18.65.7 (0–242.7)	16.6±38.25 (1–167.3)	**<0.0001**	**0.0020**	0.63	0.14
ACL graft (yes)								
Patellar tendon autograft	1276 (5.6)	1214 (5.6)	62 (5.5)	0				
Semitendinosus autograft	20 898 (92.2)	19 821 (92.2)	1041 (92.5)	36 (100.0)				
Quadriceps tendon autograft	354 (1.6)	338 (1.6)	16 (1.4)	0				
Allograft	82 (0.4)	77 (0.4)	5 (0.4)	0				
Direct suture/synthetic/other	44 (0.2)	42 (0.2)	2 (0.2)	0	0.90			
Meniscus injury (yes)								
Lateral meniscus	5557 (24.2)	5239 (24.1)	313 (27.4)	5 (13.9)	**0.015**	**0.014**	0.21	0.097
Medial meniscus	6026 (26.3)	5783 (26.6)	236 (20.6)	7 (19.4)	**<0.0001**	**<0.0001**	0.44	1.00
Cartilage injury (yes)								
Lateral femoral condyle	1257 (5.5)	1208 (5.6)	48 (4.2)	1 (2.8)	0.11	0.051	0.80	1.00
Medial femoral condyle	4216 (18.4)	4050 (18.6)	160 (14.0)	6 (16.7)	**0.0004**	**<0.0001**	0.97	0.79
Lateral patella	687 (3.0)	669 (3.1)	18 (1.6)	0	**0.0084**	**0.0024**	0.65	1.00
Medial patella	1190 (5.2)	1149 (5.3)	41 (3.6)	0	**0.015**	**0.010**	0.28	0.55
Lateral tibial plateau	1461 (6.4)	1412 (6.5)	49 (4.3)	0	**0.0034**	**0.0022**	0.18	0.42
Medial tibial plateau	1217 (5.3)	1181 (5.4)	34 (3.0)	2 (5.6)	**0.0014**	**0.0001**	1.00	0.60
Trochlea	721 (3.1)	699 (3.2)	22 (1.9)	0	**0.028**	**0.013**	0.62	1.00
Collateral ligament injury (yes)								
MCL	963 (4.2)	913 (4.2)	47 (4.1)	3 (8.3)	0.46			
LCL	232 (1.0)	225 (1.0)	7 (0.6)	0	0.32			
PLC injury (yes)	51 (0.2)	48 (0.2)	3 (0.3)	0	0.92			
Activity at the time of injury (yes)								
Alpine/skiing	3798 (16.6)	3693 (17.0)	103 (9.0)	2 (5.6)				
Pivoting sport	14 565 (63.6)	13 638 (62.8)	900 (78.7)	27 (75.0)				
Non-pivoting sport	991 (4.3)	954 (4.4)	36 (3.1)	1 (2.8)				
Other physical activity	986 (4.3)	953 (4.4)	33 (2.9)	0				
Traffic-related	409 (1.8)	396 (1.8)	11 (1.0)	2 (5.6)				
Other	2135 (9.3)	2071 (9.5)	60 (5.2)	4 (11.1)	**<0.0001**	**<0.0001**	0.14	0.063
Tibial fixation (yes)								
Interference screw	14 545 (63.4)	13 802 (63.5)	719 (62.8)	24 (66.7)				
Intratunnel fixation	473 (2.1)	457 (2.1)	16 (1.4)	0				
Suture post	5053 (22.0)	4780 (22.0)	265 (23.2)	8 (22.2)				
Retroscrew	280 (1.2)	253 (1.2)	25 (2.2)	2 (5.6)				
Fixed suspensory fixation	22 (0.1)	21 (0.1)	1 (0.1)	0				
Adjustable suspensory fixation	2555 (11.1)	2435 (11.2)	118 (10.3)	2 (5.6)	**0.022**	**0.019**	0.17	0.68
Femoral fixation (yes)								
Fixed suspensory fixation	9644 (42.1)	9099 (41.8)	531 (46.4)	14 (38.9)				
Intratunnel fixation	3817 (16.6)	3646 (16.8)	164 (14.3)	7 (19.4)				
Interference screw	3219 (14.0)	3014 (13.9)	194 (17.0)	11 (30.6)				
Adjustable suspensory fixation	6248 (27.3)	5989 (27.5)	255 (22.3)	4 (11.1)	**<0.0001**	**<0.0001**	0.012	0.080
Follow-up time after most recent surgery (years)	9.19±4.079.2 (1.21–17.14)	9.32±4.069.34 (2.2–17.14)	6.73±3.516.44 (1.21–17.05)	5.62±2.694.99 (2.35–14.04)	**<0.0001**	**0.0020**	**0.0020**	0.066

Values are given as n (%) and mean±SD or median (minimum-–maximum) for categorical and continuous as well as ordinal variables, respectively. The sums may vary to because of missing values. The variables with missing values, n (%) of the total sample were BMI 7900 (34.2), Ttime from injury to surgery 618 (2.7), and Aactivity at the time of injury 44 (0.2).

Pivoting sport (American football/rugby, basketball, dancing, floorball, gymnastics, handball, ice hockey/bandy, martial arts, racket sports, soccer, volleyball, wrestling); Nnon-pivoting sport (cross-country skiing, cycling, horseback riding, motocross/endure, skateboarding, snowboarding, and surfing/wakeboarding); Aalpine/skiing; Oother physical activity (other recreational sport, exercise, trampoline); Ttraffic related; and Oother (other outdoor activity and work). Tibial fixation devices were divided into 6six different subcategories: (1) Iinterference screw (metal screw, metal screw with backup staple fixation, resorbable screw, resorbable screw with backup post fixation, metal screw with backup osteosuture, intrafix); (2) Iintratunnel fixation (rigidfix); (3) Ssuture post (AO screw, suture washer); (4) Rretroscrew; (5) Ffixed suspensory fixation (retrobutton); (6) Aadjustable suspensory fixation (tightrope). Femoral fixation devices were also divided into four different subcategories: (1) Ffixed suspensory fixation (endobutton, retrobutton, ezloc); (2) Iintratunnel fixation (rigidfix, transfix); (3) Iinterference screw (metal screw); (4) Aadjustable suspensory fixation (toggleloc, ultrabutton).

signficant p value are in bold.

ACLanterior cruciate ligamentACLRanterior cruciate ligament reconstructionBMIbody mass indexLCLlateral collateral ligamentLMlateral meniscusMCLmedial collateral ligamentPLCposterior lateral corner

### Postoperative outcomes

**Figure 1:**
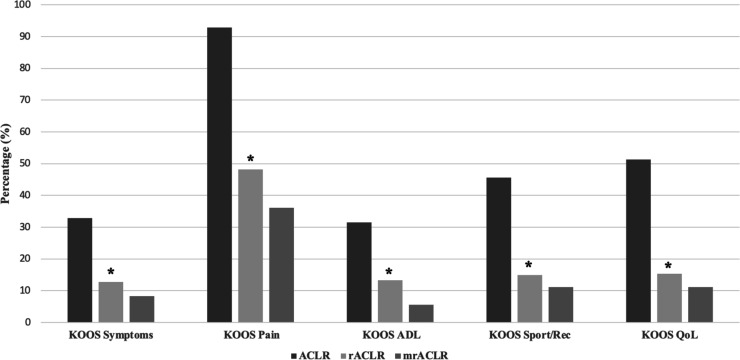
Percentages of patients meeting PASS for all KOOS subscales by procedure. *p<0.001 when comparing ACLR to rACLR. ACLR, anterior cruciate ligament reconstruction; KOOS, Knee injury and Osteoarthritis Outcome Score; mrACLR, multiple revision anterior cruciate ligament reconstruction; PASS, Patient-Acceptable Symptom State; QoL, Quality of Life; rACLR, revision anterior cruciate ligament reconstruction; Sport/Rec, Function in Sport and Recreation.

A significant difference was found in the percentage of patients achieving PASS across all KOOS subscales in patients who underwent primary ACLR versus patients who underwent rACLR (table 2 and figure 1). For example, 51.4% of patients undergoing primary ACLR surgery achieved PASS for the KOOS QoL subscale, while only 26.9% of patients treated with rACLR achieved PASS for the same subscale 1 year after the latest ACLR. No difference was found in the percentages of patients reaching PASS for all KOOS subscales between patients undergoing rACLR versus mrACLR. Mean values for the KOOS subscales in patients undergoing primary ACLR, rACLR and mrACLR can be found in online supplemental table S2. A significant difference was found in postoperative mean KOOS values in all subscales for primary ACLR compared with rACLR and mrACLR (online supplemental table S2). Finally, there were no significant predictors for achieving the PASS for the KOOS QoL and Sport/Rec subscales in patients undergoing mrACLR (tables3  4).

**Table 2:** Postoperative PASS value for the KOOS subscales

**1**-year postoperative PASS	Total (n=22 928)	Only one ACLR (n=21 748)	One revision ACLR (n=1144)	More than one revision ACLR (n=36)	P value	Only one ACLR vs one revision ACLR	Test between groups p value	
							Only one ACLR vs more than one revision ACLR	One revision ACLR vs more than one revision ACLR
KOOS Symptoms	7301 (32.6)	7152 (32.9)	146 (22.5)	3 (18.8)	**<0.0001**	**<0.0001**	0.35	1.00
KOOS Pain	20 756 (92.6)	2,0191 (92.9)	552 (84.9)	13 (81.3)	**<0.0001**	**<0.0001**	0.2	0.89
KOOS ADL	6992 (31.2)	6837 (31.4)	153 (23.5)	2 (12.5)	**<0.0001**	**<0.0001**	0.16	0.48
KOOS Sport/Rec	10 082 (45.0)	9907 (45.6)	171 (26.3)	4 (25.0)	**<0.0001**	**<0.0001**	0.16	1.00
KOOS QoL	11 353 (50.7)	11 174 (51.4)	175 (26.9)	4 (25.0)	**<0.0001**	**<0.0001**	0.06	1.00

Table presenting the postoperative PASS values for the KOOS subscales between the study groups.

signficant p value are in bold.

ACLRanterior cruciate ligament reconstructionADLActivities of Daily LivingKOOSKnee Injury and Osteoarthritis Outcome ScorePASSPatient Acceptable Symptom StateQoLQuality of LifeSport/RecFunction in Sport and Recreation

**Table 3:** Predictors of KOOS PASS QoL at 1-year follow-up

Variable	Missing	Value	PASS QoL at Follow-up	OR (95% CI)PASS KOOSQoL	P value	Area under ROC curve (95% CI)	OR (95% CI) PASS KOOS QoL	P value
Age at the time of surgery	0	20–<25	2 (50.0)					
		25–<31	2 (28.6)					
		31–50	0 (0.0)	0.21 (0.02 to 2.67)	0.23	0.73 (0.46 to 0.99)	0.07 (0.00 to 12.99)	0.32
Sex	0	Female	2 (28.6)					
		Male	2 (22.2)	0.71 (0.07 to 6.92)	0.77	0.54 (0.22 to 0.86)	0.37 (0.02 to 6.24)	0.49
BMI	8	19.6–<23.1	1 (25.0)					
		23.1–<25.8	2 (33.3)					
		25.8–34.0	1 (33.3)	0.90 (0.56 to 1.44)	0.65	0.56 (0.21 to 0.90)	0.82 (0.40 to 1.67)	0.58
Meniscus injury	0	No	3 (27.3)					
		Yes	1 (20.0)	0.67 (0.05 to 8.64)	0.76	0.54 (0.26 to 0.82)	0.37 (0.01 to 10.08)	0.56
Cartilage injury	0	No	2 (28.6)					
		Yes	2 (22.2)	0.71 (0.07 to 6.92)	0.77	0.54 (0.22 to 0.86)	1.91 (0.03 to 137.04)	0.77
Ligament/PLC injury	0	No	4 (30.8)					
		Yes	0 (0.0)	Lim (OR)=0	0.79	N/A	Lim (OR)=0	0.79
ACL graft	1	Patellar vs patellar	3 (50.0)	1.00	0.42***		1.00	0.77***
		Semitendinosus vs semitendinosus	0 (0.0)	1.00			1.00	
		Quadiceps vs patellar	0 (0.0)	0.00 (0.00–517E183)	0.96		0.00 (0.00–74E226)	0.97
		Allograft vs patellar	1 (14.3)	Lim (OR)=0		N/A	Lim (OR)=0	

All the tests were performed with univariable logistic regression and adjusted for preoperative KOOS QoL using logistic regression.

ACL, anterior cruciate ligament; BMI, body mass index; KOOSKnee injury and Osteoarthritis Outcome ScorePASS, Patient Acceptable Symptom State; PLC, posterolateral corner; QoL, Quality of Life; ROC, receiver operating characteristic

**Table 4:** Predictors of KOOS PASS Sport/Rec at 1-year follow-up

Variable	Missing	Value	PASS QoL at Follow-up	OR (95% CI)PASS KOOS Sport/Rec	P value	Area under ROC curve (95% CI)	OR (95% CI) PASS KOOS Sport/Rec	P value
Age at the time of surgery	0	20–<25	2 (50.0)					
		25–<31	2 (28.6)					
		31–50	0 (0.0)	0.21 (0.02 to 2.67)	0.23	0.73 (0.46 to 0.99)	0.00 (0.00 to 130.43)	0.27
Sex	0	Female	2 (28.6)					
		Male	2 (22.2)	0.71 (0.07 to 6.92)	0.77	0.54 (0.22 to 0.86)	0.28 (0.01 to 11.36)	0.50
BMI	8	19.6–<23.1	1 (25.0)					
		23.1–<25.8	2 (33.3)					
		25.8–34.0	1 (33.3)	0.90 (0.56 to 1.44)	0.65	0.56 (0.21 to 0.90)	1.12 (0.46 to 2.77)	0.80
Meniscus injury	0	Nej	3 (27.3)					
		Ja	1 (20.0)	0.67 (0.05 to 8.64)	0.76	0.54 (0.26 to 0.82)	0.15 (0.00 to 14.30)	0.42
Cartilage injury	0	Nej	2 (28.6)					
		Ja	2 (22.2)	0.71 (0.07 to 6.92)	0.77	0.54 (0.22 to 0.86)	11.32 (0.07 to 1914.63)	0.35
Ligament/PLC injury	0	No	4 (30.8)					
		Yes	0 (0.0)	Lim (OR)=0	0.79	N/A	Lim (OR)=0	0.79
ACL graft	1	Patellar vs patellar	3 (50.0)	1.00	0.42***		1.00	0.77***
		Semitendinosus vs semitendinosus	0 (0.0)	1.00			1.00	
		Quadiceps vs patellar	0 (0.0)	0.00 (0.00-517E183)	0.96		0.00 (0.00-42E227)	0.97
		Allograft vs patellar	1 (14.3)	Lim (OR)=0		N/A	0.72 (0.02 to 25.07)	

All the tests were performed with univariable logistic regression and adjusted for preoperative KOOS Sport/Rec using logistic regression.

ACL, anterior cruciate ligament; BMI, body mass index; PASS, Patient Acceptable Symptom State; PLC, posterolateral corner; ROC, receiver operating characteristicSport/Rec, Function in Sport and Recreation

## Discussion

The main findings of this study were that patients undergoing ACLR in the revision setting had statistically lower percentages of reaching PASS for the KOOS 1 year postoperatively than patients undergoing primary ACLR. Across all KOOS subscales, the percentages of patients meeting PASS thresholds were statistically lower for rACLR compared with primary ACLR. Furthermore, despite the lack of statistical significance most likely due to the small sample size, percentages of patients meeting PASS for all KOOS subscales 1 year postoperatively were lower for those undergoing mrACLR compared with patients with primary ACLR. Finally, percentages of patients reaching PASS thresholds for all KOOS subscales 1 year postoperatively were comparable between patients undergoing rACLR and mrACLR and no significant predictors for achieving the PASS for the KOOS QoL and Sport/Rec subscales in patients undergoing mrACLR were found.

Percentages of patients meeting PASS for KOOS subscales postoperatively after primary ACLR in this study were comparable to those in the literature.^11 15 23 24^ A 2018 cohort study reporting on factors associated with reaching PASS for the KOOS subscales 1 year postoperatively after primary ACLR found that, out of 343 patients, 57% met PASS for the KOOS Pain, 85% for the KOOS Symptoms, 40% for the KOOS ADL, 52% for the KOOS Sport/Rec and 50% for the KOOS QoL.^15^ This study found similar results after primary ACLR for some subscales, as 93% met PASS for the KOOS pain, 31% for the KOOS ADL, 46% for the KOOS Sport/Rec and 51% for the KOOs QoL. However, little data exists reporting on patients meeting postoperative PASS thresholds for the KOOS after rACLR and mrACLR. While this study does report that percentages of patients meeting PASS for the KOOS after ACLR in the revision setting is much lower than in the primary setting, it is important to consider that PASS thresholds used in this study to assess rACLR and mrACLR are still those that have been established for primary ACLR. As decreased knee functional KOOS outcomes may be expected after ACLR in the revision setting compared with the primary setting,^4 5 25^ future prospective work should consider creating PASS thresholds for the KOOS for the revision setting as more realistic expectations for knee function in this cohort should be established.

Furthermore, younger age has been reported as a known risk factor for failure after primary ACLR.^26 27^ This aligns with the current study’s findings, as patients undergoing rACLR and mrACLR were significantly younger than those undergoing primary ACLR at the time of the index procedure (23.0 vs 22.3 vs 29.0). Proposed explanations for this finding include patients of younger age having incomplete neuromuscular maturity as well as a higher likelihood to return to strenuous cutting and pivoting sports and to partake in risk-taking behaviours.^26 28^

Finally, previous work has reported factors associated with reaching PASS for the KOOS subscales after ACL injury. Studies have shown that early, operative intervention in the form of ACLR increased the chances of reaching PASS for the KOOS subscales as compared with non-surgical treatment after ACL rupture.^11 23^ Younger age, male sex, higher preoperative KOOS scores and participation in preoperative exercise plans have been reported as characteristics increasing odds of reaching KOOS PASS 1 year postoperatively after primary ACLR^15 29^ while workers’ compensation status and a diagnosis of diabetes have been linked to decreased odds of reaching KOOS PASS in the same postoperative time period.^29^ Yet, limited research exists attempting to identify predictive factors of reaching PASS for the KOOS subscales in the rACLR setting. This study attempted to predict factors associated with reaching PASS for the KOOS QoL and KOOS Sport/Rec 1 year postoperatively after mrACLR. Yet, no predictive factors were found in the analysis. This finding may be due to the small sample size of patients in the study undergoing mrACLR and subsequently reaching PASS for these KOOS subscales postoperatively. Yet, as the number of rACLRs continues to rise, it is important to understand the predictive factors associated with the most successful functional outcomes after revision surgery.

While this study does provide valuable information on percentages of large sample sizes of patients reaching acceptable symptom states after primary and rACLR, there are several limitations worth discussing. The reliance on registry data introduces potential limitations in establishing definitive cause-and-effect relationships and may result in confounding by indication. Additionally, the original validation of the KOOS instrument was conducted with patients with knee osteoarthritis, which may restrict its construct validity when assessing outcomes following ACLR.^30 31^ Also, the PASS values used in this study were originally determined for primary ACLR and may not accurately assess patients’ symptom states when applied to cohorts of patients with different characteristics, such as those in the revision setting. This may limit the possibility of finding significant differences between the groups in some of the KOOS subscales. Therefore, future studies could benefit from establishing new, more accurate PASS values for revision cases to evaluate the functional outcomes more comprehensively in patients undergoing rACLRs.

## Conclusions

Patients undergoing ACLR in the revision setting had lower rates of reaching acceptable symptom states for functional knee outcomes than those undergoing primary ACLR. However, no significant predictors for achieving the PASS for the KOOS QoL and Sport/Rec subscales in patients undergoing mrACLR were found.

## supplementary material

10.1136/bmjopen-2023-081688 online supplemental file 1

## Data Availability

Data are available upon reasonable request.

## References

[1] Gornitzky AL, Lott A, Yellin JL (2016). Sport-Specific Yearly Risk and Incidence of Anterior Cruciate Ligament Tears in High School Athletes: A Systematic Review and Meta-analysis. Am J Sports Med.

[2] Buller LT, Best MJ, Baraga MG (2015). Trends in Anterior Cruciate Ligament Reconstruction in the United States. Orthop J Sports Med.

[3] van Eck CF, Schkrohowsky JG, Working ZM (2012). Prospective analysis of failure rate and predictors of failure after anatomic anterior cruciate ligament reconstruction with allograft. Am J Sports Med.

[4] Bigouette JP, Owen EC, MARS Group (2022). Returning to Activity After Anterior Cruciate Ligament Revision Surgery: An Analysis of the Multicenter Anterior Cruciate Ligament Revision Study (MARS) Cohort at 2 Years Postoperative. Am J Sports Med.

[5] Wright RW, Gill CS, Chen L (2012). Outcome of Revision Anterior Cruciate Ligament Reconstruction: A Systematic Review. J Bone Joint Surg Am.

[6] Kvien TK, Heiberg T, Hagen KB (2007). Minimal clinically important improvement/difference (MCII/MCID) and patient acceptable symptom state (PASS): what do these concepts mean?. Ann Rheum Dis.

[7] Daste C, Abdoul H, Foissac F (2022). Patient acceptable symptom state for patient-reported outcomes in people with non-specific chronic low back pain. Ann Phys Rehabil Med.

[8] Muller B, Yabroudi MA, Lynch A (2016). Defining Thresholds for the Patient Acceptable Symptom State for the IKDC Subjective Knee Form and KOOS for Patients Who Underwent ACL Reconstruction. Am J Sports Med.

[9] Vega JF, Jacobs CA, Strnad GJ (2019). Prospective Evaluation of the Patient Acceptable Symptom State to Identify Clinically Successful Anterior Cruciate Ligament Reconstruction. Am J Sports Med.

[10] Svantesson E, Hamrin Senorski E, Webster KE (2020). Clinical outcomes after anterior cruciate ligament injury: panther symposium ACL injuryclinical outcomes consensus group. Knee Surg Sports Traumatol Arthrosc.

[11] Persson K, Bergerson E, Svantesson E (2022). Greater proportion of patients report an acceptable symptom state after ACL reconstruction compared with non-surgical treatment: a 10-year follow-up from the Swedish National Knee Ligament Registry. Br J Sports Med.

[12] Filbay SR, Roemer FW, Lohmander LS (2023). Evidence of ACL healing on MRI following ACL rupture treated with rehabilitation alone may be associated with better patient-reported outcomes: a secondary analysis from the KANON trial. Br J Sports Med.

[13] Cristiani R, Mikkelsen C, Edman G (2020). Age, gender, quadriceps strength and hop test performance are the most important factors affecting the achievement of a patient-acceptable symptom state after ACL reconstruction. Knee Surg Sports Traumatol Arthrosc.

[14] Hamrin Senorski E, Sundemo D, Svantesson E (2019). Preoperative and Intraoperative Predictors of Long-Term Acceptable Knee Function and Osteoarthritis After Anterior Cruciate Ligament Reconstruction: An Analysis Based on 2 Randomized Controlled Trials. Arthroscopy.

[15] Hamrin Senorski E, Svantesson E, Beischer S (2018). Factors Affecting the Achievement of A Patient-Acceptable Symptom State 1 Year After Anterior Cruciate Ligament Reconstruction: A Cohort Study of 343 Patients From 2 Registries. Orthop J Sports Med.

[16] Salavati M, Akhbari B, Mohammadi F (2011). Knee injury and Osteoarthritis Outcome Score (KOOS); reliability and validity in competitive athletes after anterior cruciate ligament reconstruction. Osteoarthr Cartil.

[17] Comins J, Brodersen J, Krogsgaard M (2008). Rasch analysis of the Knee injury and Osteoarthritis Outcome Score (KOOS): a statistical re-evaluation. Scand J Med Sci Sports.

[18] von Elm E, Altman DG, Egger M (2008). The Strengthening the Reporting of Observational Studies in Epidemiology (STROBE) statement: guidelines for reporting observational studies. J Clin Epidemiol.

[19] Hamrin Senorski E, Svantesson E, Engebretsen L (2019). 15 years of the Scandinavian knee ligament registries: lessons, limitations and likely prospects. Br J Sports Med.

[20] Kvist J, Kartus J, Karlsson J (2014). Results from the Swedish national anterior cruciate ligament register. Arthroscopy.

[21] Samuelsson K, Magnussen RA, Alentorn-Geli E (2017). Equivalent Knee Injury and Osteoarthritis Outcome Scores 12 and 24 Months After Anterior Cruciate Ligament Reconstruction: Results From the Swedish National Knee Ligament Register. Am J Sports Med.

[22] Roos EM, Lohmander LS (2003). The Knee injury and Osteoarthritis Outcome Score (KOOS): from joint injury to osteoarthritis. Health Qual Life Outcomes.

[23] Bergerson E, Persson K, Svantesson E (2022). Superior Outcome of Early ACL Reconstruction versus Initial Non-reconstructive Treatment With Late Crossover to Surgery: A Study From the Swedish National Knee Ligament Registry. Am J Sports Med.

[24] Patterson BE, Culvenor AG, Barton CJ (2020). Patient-Reported Outcomes One to Five Years After Anterior Cruciate Ligament Reconstruction: The Effect of Combined Injury and Associations With Osteoarthritis Features Defined on Magnetic Resonance Imaging. Arthritis Care Res (Hoboken).

[25] Wright R, Spindler K, Huston L (2011). Revision ACL reconstruction outcomes: MOON cohort. J Knee Surg.

[26] Webster KE, Feller JA, Leigh WB (2014). Younger patients are at increased risk for graft rupture and contralateral injury after anterior cruciate ligament reconstruction. Am J Sports Med.

[27] Rahardja R, Zhu M, Love H (2020). Factors associated with revision following anterior cruciate ligament reconstruction: A systematic review of registry data. Knee.

[28] Paterno MV, Schmitt LC, Ford KR (2010). Biomechanical measures during landing and postural stability predict second anterior cruciate ligament injury after anterior cruciate ligament reconstruction and return to sport. Am J Sports Med.

[29] Beletsky A, Naami E, Lu Y (2021). The Patient Acceptable Symptomatic State in Primary Anterior Cruciate Ligament Reconstruction: Predictors of Achievement. Arthroscopy J Arthrosc Relat Surg.

[30] Hansen CF, Jensen J, Odgaard A (2022). Four of five frequently used orthopedic PROMs possess inadequate content validity: a COSMIN evaluation of the mHHS, HAGOS, IKDC-SKF, KOOS and KNEES-ACL. Knee Surg Sports Traumatol Arthrosc.

[31] Comins JD, Siersma VD, Lind M (2018). KNEES-ACL has superior responsiveness compared to the most commonly used patient-reported outcome measures for anterior cruciate ligament injury. Knee Surg Sports Traumatol Arthrosc.

