# Supplementary material for: Registry-based cohort study comparing percentages of patients reaching PASS for knee function outcomes after revision ACLR compared to primary ACLR
Source: BMJ Open. 2024 Aug 9;14(8):e081688. doi: 10.1136/bmjopen-2023-081688 (PMC11331993; doi:10.1136/bmjopen-2023-081688)
Supplement: online supplemental file 1 [file bmjopen-14-8-s001.pdf]

Table S1. Baseline characteristics prior to the latest ACLR surgery.

Variable	Total (n=22,928)	Only one ACLR (n=21,748)	One revision ACLR (n=1,144)	Multiple revision ACLR (n=36)	*P*	Test between groups *P*: Only one ACLR vs One revision ACLR	Only one ACLR vs More than one revision ACLR	One revision ACLR vs More than one revision ACLR
**Age at the time of surgery (years)**	28.9 ± 10.6<br>26 (16-74)	29.1 ± 10.7<br>26 (16-74)	26.2 ± 8.1<br>24 (16-63)	28.7 ± 7.1<br>27 (20-50)	**<0.0001**	**0.0020**	0.82	0.088
**Sex (male)**	12,055 (52.6)	11,429 (52.6)	604 (52.8)	22 (61.1)	0.58			
**BMI (kg/m^2)**	24.7 ± 3.3<br>24.2 (15.1-49.8)	24.7 ± 3.3<br>24.2 (15.1-49.8)	24.5 ± 3.1<br>24.2 (17.6-37.6)	24.7 ± 3.2<br>23.5 (19.6-34)	0.42			
**Time from injury to surgery (months)**	20.6 ± 36.6<br>8.5 (0-551)	20.9 ± 37.3<br>8.5 (0-551)	15.4 ± 19.8<br>8.4 (0.2-152)	22.6 ± 26.0<br>12 (2.7-112.9)	0.20	**0.0020**	0.62	0.070
**ACL graft (yes)**								
Patellar tendon autograft	1,942 (8.6)	1,214 (5.6)	714 (63.0)	14 (40.0)				
Semitendinosus autograft	20,053 (88.5)	19,821 (92.2)	230 (20.3)	2 (5.7)				
Quadriceps tendon autograft	459 (2.0)	338 (1.6)	116 (10.2)	5 (14.3)				
Allograft	156 (0.7)	77 (0.4)	65 (5.7)	14 (40.0)				
Direct suture/synthetic/other	50 (0.2)	42 (0.2)	8 (0.7)	0 (0.0)	**<0.0001**			
**Meniscus injury (yes)**								
Lateral meniscus	5,476 (23.9)	5,239 (24.1)	235 (20.5)	2 (5.6)	**0.0008**	**0.0061**	**0.0075**	**0.029**
Medial meniscus	6,055 (26.4)	5,783 (26.6)	264 (23.1)	8 (22.2)	**0.027**	**0.0087**	0.71	1.00
**Cartilage injury (yes)**								
Lateral femoral condyle	1,336 (5.8)	1,208 (5.6)	120 (10.5)	8 (22.2)	**<0.0001**	**<.0001**	**0.0014**	0.068
Medial femoral condyle	4,398 (19.2)	4,050 (18.6)	334 (29.2)	14 (38.9)	**<0.0001**	**<0.0001**	**0.0073**	0.29
Lateral patella	728 (3.2)	669 (3.1)	56 (4.9)	3 (8.3)	**0.0006**	**0.0017**	0.20	0.53
Medial patella	1,236 (5.4)	1,149 (5.3)	81 (7.1)	6 (16.7)	**0.0004**	**0.013**	**0.022**	0.087
Lateral tibial plateau	1,532 (6.7)	1,412 (6.5)	115 (10.1)	5 (13.9)	**<0.0001**	**<0.0001**	0.16	0.60
Medial tibial plateau	1,292 (5.6)	1,181 (5.4)	105 (9.2)	6 (16.7)	**<0.0001**	**<0.0001**	0.025	0.23
Trochlea	788 (3.4)	699 (3.2)	83 (7.3)	6 (16.7)	**<0.0001**	**<0.0001**	**0.0019**	0.096
**Collateral ligament injury (yes)**								
MCL	936 (4.1)	913 (4.2)	21 (1.8)	2 (5.6)	**0.0004**			
LCL	236 (1.0)	225 (1.0)	9 (0.8)	2 (5.6)	**0.019**			
**PLC injury (yes)**	62 (0.3)	48 (0.2)	12 (1.0)	2 (5.6)	**<0.0001**			
**Activity at the time of injury (yes)**								
Alpine/skiing	3,768 (16.5)	3,693 (17.0)	74 (6.5)	1 (2.8)				
Pivoting sport	14,361 (62.8)	13,638 (62.8)	703 (61.6)	20 (55.6)				
Non-pivoting sport	990 (4.3)	954 (4.4)	36 (3.2)	0				
Other physical activity	1,013 (4.4)	953 (4.4)	57 (5.0)	3 (8.3)				
Traffic	407 (1.8)	396 (1.8)	10 (0.9)	1 (2.8)				
Other	2,343 (10.2)	2,071 (9.5)	261 (22.9)	11 (30.6)	**<0.0001**	**<0.0001**	**0.0002**	0.40
**Tibial fixation (yes)**								
Interference screw	14,703 (64.1)	13,802 (63.5)	870 (76.5)	31 (86.1)				
Intratunnel fixation	457 (2.0)	457 (2.1)	0 (0.0)	0 (0.0)				
Suture post	4,885 (21.3)	4,780 (22.0)	105 (9.2)	0 (0.0)				
Retroscrew	265 (1.2)	253 (1.2)	12 (1.1)	0 (0.0)				
Fixed suspensory fixation	21 (0.1)	21 (0.1)	0 (0.0)	0 (0.0)				

Adjustable suspensory fixation	2,537 (11.1)	2,435 (11.2)	99 (8.7)	3 (8.3)				
Staple	38 (0.2)	0 (0.0)	38 (3.3)	0 (0.0)				
Endobutton	4 (0.0)	0 (0.0)	4 (0.4)	0 (0.0)				
Infinitybutton	1 (0.0)	0 (0.0)	1 (0.1)	0 (0.0)				
Annan	10 (0.0)	0 (0.0)	8 (0.7)	2 (5.6)	**<0.0001**	**<0.0001**	**<0.0001**	**0.034**
**Femoral fixation (yes)**	45 (0.2)	0 (0.0)	44 (3.1)	1 (2.4)				
Fixed suspensory fixation	9,340 (40.8)	9,099 (41.8)	232 (20.5)	9 (25.0)				
Intratunnel fixation	3,659 (16.0)	3,646 (16.8)	12 (1.1)	1 (2.8)				
Interference screw	3,576 (15.6)	3,014 (13.9)	550 (48.5)	12 (33.3)				
Adjustable suspensory fixation	6,324 (27.6)	5,989 (27.5)	324 (28.6)	11 (30.6)				
	2 (0.0)	0 (0.0)	2 (0.2)	0 (0.0)				
AO-screw	9,340 (40.8)	9,099 (41.8)	232 (20.5)	9 (25.0)				
Metall screw / Endopearl	2 (0.0)	0 (0.0)	2 (0.2)	0 (0.0)				
XO-button	1 (0.0)	0 (0.0)	1 (0.1)	0 (0.0)				
Infinity button	1 (0.0)	0 (0.0)	1 (0.1)	0 (0.0)				
Other	13 (0.1)	0 (0.0)	10 (0.9)	3 (8.3)	**<0.0001**	**<0.0001**	**<0.0001**	**0.0082**

Values are given as n (%) and mean ± SD or median (minimum-maximum) for categorical and continuous as well as ordinal variables, respectively. The sums may vary to because of missing values. The variables with missing values, n (%) of the total sample were BMI 7,846 (34.2), Time from injury to surgery 679 (3.0), ACL graft 268 (1.2), Activity at the time of injury 46 (0.3), Tibial fixation 7 (0.03), and Femoral fixation 10 (0.04). ACL=anterior cruciate ligament; ACLR=anterior cruciate ligament reconstruction; BMI=body mass index; LCL=lateral collateral ligament; LM=lateral meniscus; MCL=medial collateral ligament; PLC=posterior lateral corner; SD=standard deviation.

Pivoting sport (American football/rugby, basketball, dancing, floorball, gymnastics, handball, ice hockey/bandy, martial arts, racket sports, soccer, volleyball, wrestling); Non-pivoting sport (cross-country skiing, cycling, horseback riding, motocross/endure, skateboarding, snowboarding, and surfing/wakeboarding); Alpine/skiing; Other physical activity (other recreational sport, exercise, trampoline); Traffic related; and Other (other outdoor activity and work).Tibial fixation devices were divided into 6 different subcategories: 1) Interference screw (metal screw, metal screw with backup staple fixation, resorbable screw, resorbable screw with backup post fixation, metal screw with backup osteosuture, intrafix); 2) Intratunnel fixation (rigidfix); 3) Suture post (AO screw, suture washer); 4) Retroscrew; 5) Fixed suspensory fixation (retrobutton); 6) Adjustable suspensory fixation (tightrope). Femoral fixation devices were also divided into four different subcategories: 1) Fixed suspensory fixation (endobutton, retrobutton, ezloc); 2) Intratunnel fixation (rigidfix, transfix); 3) Interference screw (metal screw); 4) Adjustable suspensory fixation (toggleloc, ultrabutton).

Table S2. 1-year postoperative KOOS

Variable	Total (n=22,928)	Only one ACLR (n=21,748)	One revision ACLR (n=1,144)	More than one revision ACLR (n=36)	*P*	Test between groups *P*<br>Only one ACLR vs One revision ACLR	Only one ACLR vs More than one revision ACLR	One revision ACLR vs More than one revision ACLR
**KOOS Symptoms**	76.5 ± 18.0<br>78.6 (0-100)<br>n=22,410	76.5 ± 17.9<br>78.6 (0-100)<br>n=21,744	70.7 ± 20.1<br>75 (0-100)<br>n=650	60.5 ± 23.9<br>58.9 (17.9-100)<br>n=16	**<0.0001**	**<0.0001**	**0.0050**	0.073
**KOOS Pain**	84.0 ± 15.7<br>88.9 (0-100)<br>N=22,410	84.2 ± 15.5<br>88.9 (0-100)<br>n=21,744	77.7 ± 19.2<br>83.3 (5.6-100)<br>n=650	65.8 ± 26.8<br>70.8 (5.6-94.4)<br>n=16	**<0.0001**	**<0.0001**	**0.0008**	0.051
**KOOS ADL**	91.1 ± 13.3<br>97.1 (0-1009<br>n=22,411	91.2 ± 13.2<br>97.1 (0-100)<br>n=21,745	87.1 ± 17.2<br>94.1 (2.9-100)<br>n=650	79.3 ± 23.7<br>89 (22.1-100)<br>n=16	**<0.0001**	**<0.0001**	**0.011**	0.12
**KOOS Sport/Rec**	63.7 ± 26.9<br>70 (0-100)<br>n=22,405	64.1 ± 26<br>70 (0-100)<br>n=21,739	51.1 ± 28.6<br>52.2 (0-100)<br>n=650	44.1 ± 30.7<br>42.5 (0-95)<br>n=16	**<0.0001**	**<0.0001**	**0.0079**	0.31
**KOOS QoL**	58.1 ± 23.1<br>62.5 (0-100)<br>n=22,400	58.6 ± 23.0<br>62.5 (0-100)<br>n=21,734	44.8 ± 23.7<br>43.8 (0-100)<br>n=650	41.8 ± 23.4<br>37.5 (0-81.3)<br>n=16	**<0.0001**	N/A	N/A	0.65

Values are given as n (%) and mean ± SD or median (minimum-maximum) for categorical and continuous as well as ordinal variables, respectively. The sums may vary to because of missing values.
ADL=activities of daily living; KOOS=knee injury and osteoarthritis outcome score; QoL=quality of life; Sport/Rec=Function in sport and recreation.
