# Supplementary material for: Registry-based cohort study comparing percentages of patients reaching PASS for knee function outcomes after revision ACLR compared to primary ACLR
Source: BMJ Open. 2024 Aug 9;14(8):e081688. doi: 10.1136/bmjopen-2023-081688 (PMC11331993; doi:10.1136/bmjopen-2023-081688)
Supplement: Supplementary file 2 [file bmjopen-2023-081688.reviewer_comments.pdf]

PEER REVIEW HISTORY

BMJ Open publishes all reviews undertaken for accepted manuscripts. Reviewers are asked to complete a checklist review form and are provided with free text boxes to elaborate on their assessment. These free text comments are reproduced below.

ARTICLE DETAILS

**TITLE (PROVISIONAL)**	A Registry-Based Cohort Study Comparing Percentages of Patients Reaching PASS for Knee Function Outcomes after Revision ACLR Compared to Primary ACLR
**AUTHORS**	Herman, Zachary; Kaarre, Janina; Grassi, Alberto; Senorski, Eric; Musahl, Volker; Samuelsson, Kristian

VERSION 1 - REVIEW

**REVIEWER NAME**	Jeyaraman, Madhan
**REVIEWER AFFILIATION**	Sharda University
**REVIEWER CONFLICT OF INTEREST**	No competing interests
**DATE REVIEW RETURNED**	03-Feb-2024

**GENERAL COMMENTS**	This manuscript enrol a large number of participants to compare revision ACLR and primary ACLR. This study imparts a landmark in scientific literature by helping clinicians in treating revision ACL reconstruction

**REVIEWER NAME**	von Essen, Christoffer
**REVIEWER AFFILIATION**	Karolinska Institute, Orthopedic Surgery
**REVIEWER CONFLICT OF INTEREST**	None
**DATE REVIEW RETURNED**	04-Apr-2024

**GENERAL COMMENTS**	Thank you for the opportunity to review the paper<br>I thank the authors for their work and efforts in performing this study.<br>The article describes outcomes after revision surgery compared to primary surgery. This is a topic on which much has already been written and although this is a well-written study, there is not much that is new and draw conclusions from 36 mrACLR is that can give statistical errors<br>The manuscript has been written in a structured manner and was easy to read. English is good.<br>Title;<br>No comment<br>Abstract<br>The abstract contains the information necessary for an abstract and is less than 350 words, and such confers to the guidelines.<br>Please explain abbrevations.<br>Intoduction<br>The introduction contains all the information necessary for an introduction. You write that there is little data about rACLR, but you do not refer to any studies or how the outcome is. This should be included in the introduction, otherwise the study will be misleading,

	as there are relatively many articles about at least PASS around rACLR.<br>Method<br>Well written and contains all necessary information.<br>Although the article by Samuelsson shows that KOOS is equivalent to 12 and 24 months, how many active athletes are back in return to performance within 12 months, especially in a revision setting where we see that it takes time to recover.<br>Results<br>No comment<br>Discussion<br>Line 240; It's actually largely the same material...<br>References<br>No comment<br>Tables<br>No comment<br>Figure<br>No comment

**REVIEWER NAME**	Thompson, Xavier D.
**REVIEWER AFFILIATION**	University of Virginia
**REVIEWER CONFLICT OF INTEREST**	I also publish research related to ACL reconstruction and am a part of an ACL research consortium in the United States.
**DATE REVIEW RETURNED**	17-Apr-2024

**GENERAL COMMENTS**	Overall, I thank the authors for their contribution. I believe this is an important topic to explore and this paper will be valuable to the literature, but will be strengthened with some revisions.<br><br>Abstract<br>Objectives- Can you please provide some context for why this is important? What are PASS scores?<br>Line 61- You have not defined these acronyms yet in the abstract<br>Line 62- Is there a reason for examining these subscales specifically?<br>Line 80- I believe this is the first time you have explicitly mentioned acceptable symptom state. A reader less familiar with PASS may not have had this context.<br><br>Introduction<br>Line 96- Are there any findings to the contrary?<br>Lines 94-98- Can you expand upon the scope of the problem and also why you hypothesize mrACLR will be different? In general what differences are there in the short and long term between these groups?<br>Line 100 Is PASS itself a one item criteria or were the thresholds developed from one?<br>Line 107 All subscales?<br>Line 108 How is clinical success defined?<br>Line 106-111 I recommend splitting this into two sentences.<br>Line 111-112 Why is it valuable? Does it simplify or guide decision making?<br>Line 120 You have not discussed why these specific subscales are important<br>In the introduction, it would be helpful to discuss the specific subscales of the KOOS in some form if you are going to mention them in your purpose. You should also discuss your patient population in greater detail. As it stands your introduction is very

	brief and leaves space for you to provide additional information. In short how does this matter in the context of clinical care or decision making?<br><br><br>Methods<br>IRB or consent information?<br>Line 172-173 Did you consider a sub analysis only including 1-year data?<br>Line 173-175 Move this to after you discuss the subscales.<br>Either here or in the introduction you should discuss who the PASS scores were developed on<br>Statistical Analysis- Your use of nonparametric statistics seems appropriate, but given your large sample size did you consult a bio statistician? My concern is with a sample so large you are more likely to find a significant result.<br>After reading your discussion where is the prediction data analysis coming in?<br>Results<br>Line 203- Even with nonparametric statistics such large differences in group sizes is concerning. Write out how many primary.<br>Line 206- It would be helpful to add here or in Table 1 Time from injury to Index ACLR. Also, what about age at the time of this surgery?<br>Line 214- List them all or reference table. Speak about the differences for each subscale and direction by group.<br>Line 221- Can you be more specific about direction?<br>Line 234- Is there also a consideration that a rather large sample size influenced statistical significance?<br>Line 256-259 This confuses me. Is this not just a function of the primary group having their first injury later? What does this have to do with failure age? I am interpreting index injury as their first ACLR ever.<br>Line 260 I think this is the first mention of return to sport. While important you should bring this up in the introduction.<br>Conclusions<br>Do not forget your second aim.<br>Table 2.<br>Can you further explain this table in the caption?<br>Table 4.<br>Where did this ROC curve come from? I do not recall a discussion of this in the methods.<br>It appears that you have two Table 2’s. I am not sure which is supplemental based on titles.<br>Also, some of the p’s in your tables appear capitalized while others are not.

VERSION 1 – AUTHOR RESPONSE

Reviewer: 1
Dr. Madhan Jeyaraman, Sharda University
Comments to the Author:

This manuscript enrol a large number of participants to compare revision ACLR and primary ACLR. This study imparts a landmark in scientific literature by helping clinicians in treating revision ACL reconstruction

Reviewer: 2
Dr. Christoffer von Essen, Karolinska Institute

Comments to the Author:
Thank you for the opportunity to review the paper
I thank the authors for their work and efforts in performing this study. The article describes outcomes after revision surgery compared to primary surgery. This is a topic on which much has already been written and although this is a well-written study, there is not much that is new and draw conclusions from 36 mrACLR is that can give statistical errors
The manuscript has been written in a structured manner and was easy to read. English is good.

Title;
No comment

Abstract
The abstract contains the information necessary for an abstract and is less than 350 words, and such confers to the guidelines.
Please explain abbrevations.

Response: We have changed accordingly. Please see the abstract.

Intoduction
The introduction contains all the information necessary for an introduction. You write that there is little data about rACLR, but you do not refer to any studies or how the outcome is. This should be included in the introduction, otherwise the study will be misleading, as there are relatively many articles about at least PASS around rACLR.

Response: Please see lines “181-182” “Patient-reported knee function and rate of return to sport after rACLR is widely accepted as inferior to that following the primary procedure..”

Method
Well written and contains all necessary information.
Although the article by Samuelsson shows that KOOS is equivalent to 12 and 24 months, how many active athletes are back in return to performance within 12 months, especially in a revision setting where we see that it takes time to recover.

Response: Thank you for your comment. While this is true, we believe it is important to determine whether these patients were able to meet PASS thresholds for KOOS subslaces, as this information can also enhance our understanding of the differences in clinical success after surgery and the ability to return the sports at 1 year postoperation.

Results
No comment

Discussion
Line 240; It's actually largely the same material...

Response: Thank you for your comment. However, we felt it was important to compare our study results to previous studies. It is interesting to note that no large changes have occurred in the

intervening years.

References
No comment

Tables
No comment

Figure
No comment

Reviewer: 3
Dr. Xavier D. Thompson, University of Virginia
Comments to the Author:
Overall, I thank the authors for their contribution. I believe this is an important topic to explore and this paper will be valuable to the literature, but will be strengthened with some revisions.

Abstract

Objectives- Can you please provide some context for why this is important? What are PASS scores?

Response: We have revised accordingly. Please see lines “87-90“
“Reaching the Patient-Acceptable Symptom State (PASS) threshold for the Knee injury and Osteoarthritis Outcome Score (KOOS) has previously been reported to successfully identify individuals experiencing clinical success after anterior cruciate ligament reconstruction (ACLR).”

Line 61- You have not defined these acronyms yet in the abstract

Response: Thank you for your comment. We have changed accordingly.

Line 62- Is there a reason for examining these subscales specifically?

Response: Thank you for your question. Yes, we believe that this data is for understanding possible differences in the different subscales between the groups. Thus, it is possible that while differences may not be found in one KOOS subscale, disparities can be seen in other KOOS subscales. E.g., patients with several revisions may experience more Pain, while Function in Sport and Recreation remains the same between the groups ◊ increased knowledge.

Line 80- I believe this is the first time you have explicitly mentioned acceptable symptom state. A reader less familiar with PASS may not have had this context.

Response: Thank you for your comment. We have explained “the PASS” at the beginning of the abstract. Please see line “87”. “Reaching the Patient-Acceptable Symptom State (PASS)…”

Introduction

Line 96- Are there any findings to the contrary?

Response: There may be studies reporting similar outcomes. However, most of the current literature reports inferior outcomes following rACLR compared to primary ACLR. However, the outcomes vary depending on the patient age and activity level as less active older individuals may not require the

same knee function as a younger elite athlete.

Lines 94-98- Can you expand upon the scope of the problem and also why you hypothesize mrACLR will be different? In general what differences are there in the short and long term between these groups?

Response: Thank you for your comment. We have clarified this by explaining why the increased understanding of the percentages and predictors of meeting PASS between the groups is important. Please see lines 210-213" "Thus, this information would be particularly important for a better understanding of patient outcomes and counseling patients before their respective surgery about their future subjective QoL and knee function."

While we appreciate the question regarding short- and long-term outcomes, the goal of the study was to focus solely on the short-term outcomes. Therefore, we believe that discussing the long-term outcomes in the introduction may confuse the readers. However, if the Editor wishes us to add this information, we would be happy to do so.

Line 100 Is PASS itself a one item criteria or were the thresholds developed from one?

Response: PASS is a one-item criterion. More detailed information can be found in the methods sections.

Line 107 All subscales?

Response: Please see below.

Line 108 How is clinical success defined?

Response: We added a sentence about how Vega et al., had defined the clinical success in their study. Please see lines ""
"Vega et al. defined clinical success in their study as "changes in preoperative scores on the KOOS Pain subscale and the KOOS Knee Related Quality of Life (Qol) subscale in excess of minimally important difference or final KOOS Pain or QoL subscale scores in excess of previously defined PASS thresholds."

Line 106-111 I recommend splitting this into two sentences.

Response: We have changed accordingly. Please see lines "191-204"
"In fact, reaching the PASS threshold for Knee injury and Osteoarthritis Outcome Score (KOOS) has successfully identified individuals who have experienced clinical success after ACLR with high sensitivity. Vega et al. defined clinical success in their study as "changes in preoperative scores on the KOOS Pain subscale and the KOOS Knee Related Quality of Life (Qol) subscale in excess of minimally important difference or final KOOS Pain or QoL subscale scores in excess of previously defined PASS thresholds."A 2020 ACLR consensus group concluded that measurement of PASS is valuable in the assessment of outcomes after ACLR and should be a priority, as the question of whether a patient perceives an acceptable symptom state is important."

Line 111-112 Why is it valuable? Does it simplify or guide decision making?

Response: We have clarified this as suggested. Please see lines "204-206" "Furthermore, it is agreed upon that PASS values provide a tool to assist clinicians in the interpretation of patient-reported outcomes, thus, PASS values can assist clinical decision making."

Line 120 You have not discussed why these specific subscales are important
In the introduction, it would be helpful to discuss the specific subscales of the KOOS in some form if you are going to mention them in your purpose. You should also discuss your patient population in greater detail. As it stands your introduction is very brief and leaves space for you to provide additional information. In short how does this matter in the context of clinical care or decision making?

Response: Thank you for your comment. We have made the suggested changes. Please see previous responses and lines "217-220" "Secondarily, it sought to examine the injury-, patient-, and surgery-related predictors for reaching PASS for KOOS QoL and Function in Sport and Recreation (Sport/Rec) after mrACLR as these subscales have previously been recognized as viable subscales for assessing knee function and QoL in patient undergoing ACLR."

Methods
IRB or consent information?

Response: Thank you for your comment. The study had IRB approval approved by the Regional Ethical Board in Stockholm, Sweden (2011/337-31/3), and the Swedish Ethical Review Authority (2022-00913-01). The Swedish National Knee Ligament Registry complies with the Swedish legislation related to data security and therefore, no informed consent is necessary for national databases in Sweden. Investigators had access only to unidentifiable patient data.

Line 172-173 Did you consider a sub-analysis only including 1-year data?

Response: Thank you for your question. We did not consider performing sub-analyses as that was not the main goal of this study, and they could subsequently diminish the value of the study.

Line 173-175 Move this to after you discuss the subscales.
Either here or in the introduction you should discuss who the PASS scores were developed on

Response: We have made the suggested changes. Please see lines "291-294". "Additionally, the predictors for KOOS Function in Sport and Recreation (Sport/Rec), and Quality of Life (QoL) subscales were evaluated in patients undergoing mrACLR. The KOOS questionnaire comprises five subscales, namely Pain, Symptoms, Sport/Rec, QoL and Activities of Daily Living (ADL)."

And lines "295-296". "The pass cutoffs (sensitivity; specificity) for patients undergoing ACLR have been previously reported to be.."

Statistical Analysis- Your use of nonparametric statistics seems appropriate, but given your large sample size did you consult a bio statistician? My concern is with a sample so large you are more likely to find a significant result.

Response: Thank you for your comments. All the analyses were performed by statisticians as stated in the acknowledgments section.

After reading your discussion where is the prediction data analysis coming in?

Response: Please see lines ".367-370" "Lastly, percentages of patients reaching PASS thresholds for all KOOS subscales 1 year postoperatively was comparable between patients undergoing rACLR and mrACLR and no significant predictors for achieving the PASS for the KOOS QoL and Sport/Rec subscales in patients undergoing mrACLR were found."

Results

Line 203- Even with nonparametric statistics such large differences in group sizes is concerning. Write out how many primary.

Response: We have made the suggested changes. Please see lines “332-333”.
“Out of the 22,928 patients who were included in the study, 21,748 underwent primary ACLR 1,144 underwent rACLR and 36 underwent mrACLR (Table 1).”

Line 206- It would be helpful to add here or in Table 1 Time from injury to Index ACLR. Also, what about age at the time of this surgery?

Response: Table 1 includes information from “injury to index surgery”. (row 8)

Table 1 includes “age at the time of surgery” before the index ACLR (row 3) and Supplementary Table S1 includes information on “age at the time of surgery” before the latest ACLR surgery (row 3)

Line 214- List them all or reference table. Speak about the differences for each subscale and direction by group.

Response: Thank you for your comment. This information is referred to Table 2 and Figure 1. Please see lines “343-345” “A significant difference was found in the percentage of patients achieving PASS across all KOOS subscales in patients who underwent primary ACLR versus patients who underwent rACLR (Table 2 and Figure 1).”

Line 221- Can you be more specific about direction?

Response: Thank you for your comment. We have changed accordingly. Please see lines “349-350”.
“Mean values for KOOS subscales in patients undergoing primary ACLR, rACLR, and mrACLR can be found in the tables in the supplemental material (Table S2).”

Line 234- Is there also a consideration that a rather large sample size influenced statistical significance?

Response: Thank you for your comment. This is a good point and may be possible. However, we believe it is most likely that a small sample size has influenced the results, as there were not that many mrACLR patients with KOOS data.

Line 256-259 This confuses me. Is this not just a function of the primary group having their first injury later? What does this have to do with failure age? I am interpreting index injury as their first ACLR ever.

Response: Thank you for your question. We believe this is an important finding. The patients who later underwent revision or multiple revision ACLR were significantly younger at the time of primary ACLR (index ACLR). This finding could explain why these patients also underwent revision surgery, as the younger at the time of ACLR is considered a risk factor for revision ACLR. In contrast, patients who underwent primary ACLR were older and may therefore have engaged in less knee-strenuous activities, potentially protecting them from revision surgery.

Line 260 I think this is the first mention of return to sport. While important you should bring this up in the introduction.

Response: We have included this in the introduction. Please see line”181-182” “Patient-reported knee function and rate of return to sport after rACLR is widely accepted as inferior to that following the primary procedure…”

Conclusions

Do not forget your second aim.

Response: Thank you for your comment. We have updated the conclusion accordingly. Please see lines “446-448” “However, no significant predictors for achieving the PASS for the KOOS QoL and Sport/Rec subscales in patients undergoing mrACLR were found.”.

Table 2.
Can you further explain this table in the caption?

Response: We have updated accordingly. Please see the table captions. “Table demonstrating the comparison of postoperative PASS values for the KOOS subscales between the study groups.”

Table 4.
Where did this ROC curve come from? I do not recall a discussion of this in the methods.

Response: We have updated accordingly. Please see the methods and table captions.
Lines “325-326”. “Finally, univariable logistic regression analyses were used for predictor analyses and presented as odds ratio (OR) and area under the receiver operating characteristic (ROC).”
Table 3. Line “598” “All the tests were performed with univariable logistic regression and adjusted for preoperative KOOS QoL using logistic regression.”
Table 4. Lines”605” “All the tests were performed with univariable logistic regression and adjusted for preoperative KOOS Sport/Rec using logistic regression.”

It appears that you have two Table 2’s. I am not sure which is supplemental based on titles.

Response: We have updated the titles for the supplementary tables and indicated them as “S1” and “S2”, respectively.

Also, some of the p’s in your tables appear capitalized while others are not.

Response: We have updated accordingly.

Reviewer: 1
Competing interests of Reviewer: No competing interests

Reviewer: 2
Competing interests of Reviewer: none

Reviewer: 3
Competing interests of Reviewer: I also publish research related to ACL reconstruction and am a part of an ACL research consortium in the United States.

VERSION 2 – REVIEW

**REVIEWER NAME**	Thompson, Xavier D.
**REVIEWER AFFILIATION**	University of Virginia
**REVIEWER CONFLICT OF INTEREST**	I also publish research related to ACL reconstruction and am a part of an ACL research consortium in the United States.
**DATE REVIEW RETURNED**	29-Jun-2024

**GENERAL COMMENTS**	Thank you for addressing my comments. I have nothing further to add.

VERSION 2 – AUTHOR RESPONSE
